# The impact of breast cancer on social cognition in female Colombian patients

**DOI:** 10.1186/s40359-022-01005-1

**Published:** 2022-12-13

**Authors:** Nicole Palacio, Daniela Nicole Romero, Andrés Mateo Bernal, Daniela González-Rodríguez, Daniel Solarte-Bothe, María del Pilar García, Raúl Murillo, Hernando Santamaría-García, Sandra Báez

**Affiliations:** 1 Departamento de Psicología, Universidad de Los Andes, Carrera 1 # 18A-12, 111711 Bogotá, Colombia; 2 Integrated Program in Neuroscience, McGill University, Montreal, Canada; 3 Master’s Program Psychological Research, Texas State University, Texas, USA; 4 Centro de Memoria y Cognición Intellectus, Hospital Universitario San Ignacio, Bogotá, Colombia; 5 Centro Javeriano de Oncología, Hospital Universitario San Ignacio, Bogotá, Colombia; 6 Facultad de Medicina, Pontificia Universidad Javeriana, Bogotá, Colombia; 7 Doctorado en Neurociencias, Pontificia Universidad Javeriana, Bogotá, Colombia

**Keywords:** Breast cancer, Cancer, Emotion recognition, Executive functions, Social cognition, Theory of mind

## Abstract

**Background:**

The high prevalence of female breast cancer is a global health concern. Breast cancer and its treatments have been associated with impairments in general cognition, as well as structural and functional brain changes. Considering the social challenges that some of these patients face, it is important to understand the socio-emotional effects of breast cancer as well. Nevertheless, the impact of breast cancer on social cognition has remained underexplored. The objective of this study was to assess social cognition domains and other relevant cognitive and emotional variables (executive functions, anxiety, or depression) in females with breast cancer.

**Methods:**

The participants were 29 female patients diagnosed with breast cancer and 29 female healthy controls. We assessed emotion recognition, theory of mind, empathy, and moral emotions. We also included measures of general cognitive functioning, quality of life, anxiety, and depression. Linear multiple regressions were performed to assess whether the group (patients or controls), GAD-7 scores, emotional and social subscales of EORTC QLQ-C30, and IFS scores predicted the social cognition variables (EET, RMET, MSAT).

**Results:**

Patients with breast cancer showed impairments in emotion recognition and in affective theory of mind. In addition, patients had lower scores in some executive functions. Only theory of mind between group differences remained significant after Bonferroni correction. Emotion recognition was associated with executive functioning, but anxiety levels were not a significant predictor of the changes in social cognition.

**Conclusions:**

Social cognition impairments, especially in theory of mind, may be present in breast cancer, which can be relevant to understanding the social challenges that these patients encounter. This could indicate the need for therapeutic interventions to preserve social cognition skills in patients with breast cancer.

**Supplementary Information:**

The online version contains supplementary material available at 10.1186/s40359-022-01005-1.

## Background

Breast cancer is the most frequently diagnosed female cancer and it has become an important cause of death [1]. It can also affect multiple psychological domains, including cognition and emotion. Anxiety, body image alterations, and depression are relatively common health concerns in some breast cancer patients [2, 3] and breast cancer survivors can still face anxiety in the form of fear of cancer recurrence [4]. It is likely that breast cancer also affects social cognition, which could partially account for the difficulties that patients face in social networks and support. Nevertheless, this topic has been underexplored. Only a few studies have indicated that these patients experience alterations in the theory of mind (ToM) [5–7]. Thus, there is a significant knowledge gap regarding the social cognition domains that may be compromised. An improved understanding of social cognition could contribute to better treatments to address social challenges. This could help patients to maintain or develop social interactions. Therefore, the objective of this study was to characterize and compare social cognition domains (basic emotion recognition, empathy, ToM, and moral emotions) between female breast cancer patients and healthy controls, exploring possible relationships with other relevant emotional and cognitive variables (e.g., executive functions, quality of life, and anxiety).

Cancer has pervasive effects in multiple domains. For example, it can lead to changes in the patient’s new representation of self, which can also impact their mental health and daily activities [8]. Patients also have alterations in autobiographical memories and depressive symptoms [9]. Moreover, executive functions, attention, and memory are commonly affected by chemotherapy, although cognitive impairment can also occur in other types of treatments [10]. In particular, breast cancer patients and survivors report cognitive problems [11, 12], which mainly happen in a subgroup of patients [13]. However, it should be noted that there might already be changes in cognitive performance even before adjuvant treatment [14] and cognitive alterations prior to chemotherapy can partially account for the deficits [15].

In cancer patients, the cognitive changes are partly due to cancer itself but also because of its treatments and comorbidities [16]. Although there is variation among studies, approximately 22% of invasive breast cancer patients before adjuvant treatment already have decreased cognitive performance [14]. Cancer-related cognitive impairment is even more prevalent during treatment [17] and the self-reported cognitive complaints are also common in survivors [18]. However, the cognitive decline is not observed in all patients, but particularly among those with treatment-induced menopause [19] or high-dose chemotherapy [20]. Cancer treatments can affect cognitive domains such as verbal memory [21, 22], but a meta-analysis showed that visuospatial and verbal abilities were the only ones affected at least six-months after chemotherapy [23]. Treatments without chemotherapy can also affect cognitive domains. Although more studies are needed, in some patients, hormone therapy may potentially be related to cognitive impairment [24] and, in particular, problems in processing speed and verbal memory [25].

Cancer treatments affect the brain structurally and functionally. Chemotherapy can lead to lower gray matter density in subcortical and cortical regions, particularly in the hippocampus and in the frontal and temporal areas [26]. Moreover, it has been associated with abnormalities in white matter structures, cerebral blood flow, and resting-state functional connectivity [26, 27]. Some of these changes can persist over nine years after treatment [28]. Although chemotherapy has been consistently associated with structural brain abnormalities [29], patients without chemotherapy may also have diffuse decreases in white and gray matter volumes [30]. Therefore, both cancer and its treatment could impact the structure and function of regions that are relevant for social cognition.

Breast cancer and its treatments could alter the activation and connectivity of multiple brain areas, including the prefrontal cortex [31, 32], which is relevant for social cognition tasks [33, 34]. Only a few studies have investigated social cognition in patients with breast cancer showing that they have ToM impairments [5–7]. However, other domains could also be affected because there is an overlap in the neuroanatomical substrate of several social cognition tasks [35]. In other types of cancer, like primary CNS lymphoma, patients experienced social cognition deficits even though they were in remission [36]. Thus, there is promising evidence that social cognition may be altered in cancer patients, which would subsequently impact their life and social functioning.

Social cognition alterations could potentially affect the social support system as well. There are different types of social support, including problem-oriented, daily, instrumental, and social-emotional support [37]. Different forms of support can have a beneficial impact in the patients’ quality of life and emotional well-being [38]. Although positive social support could help patients, negative social support may be detrimental (e.g., when a caregiver “takes over”) [39]. In the context of breast cancer, higher depression and stress can be associated with a lower quality of social support [40]. Taking into account the multidimensional nature of social support and the difficulties that these patients may experience [41], it is important to understand their social cognition abilities.

Currently, there is a knowledge gap regarding social cognition in the context of breast cancer. To bridge such a gap, our purpose was to evaluate several social cognition domains (basic emotion recognition, empathy, ToM, and moral emotions) in a group of female breast cancer patients, in comparison with a healthy control group. For a more comprehensive assessment, we included general cognitive tests, as well as measurements of depression, anxiety, and quality of life. We hypothesized that both general cognition and social cognition are impaired in breast cancer patients. Considering that there are similar neuroanatomical substrates behind different social cognition domains [35], we expected to find a lower performance of patients in emotion recognition, ToM, and emotional response in moral and non-moral scenarios.

## Methods

### Study design and participants

The study included 58 participants, 29 patients diagnosed with breast cancer, and 29 healthy controls. Based on a sensitivity analysis using G*Power 3.1 [42, 43] assuming an alpha of 0.05 and a power of 0.80, our sample was sufficient to detect a medium-large effect size (effect size d = 0.766 for two-tail non-parametric tests; effect size d = 0.748 for two-tail t-tests). We only included females due to the very high incidence of breast cancer in females compared to males [1, 44, 45]. Patients were recruited from the Oncology Center of the Hospital Universitario San Ignacio in Bogotá, Colombia. They were at various stages of breast cancer and could have experienced different treatments, such as chemotherapy, radiotherapy, hormonal treatment, surgery, or a combination of those, among other options (Table 1).

**Table 1:** Number of patients classified by type of treatment and breast cancer stage

Treatment	Number of patients
Chemotherapy, radiotherapy, hormone therapy, and surgery	3
Chemotherapy, hormone therapy, and surgery	1
Chemotherapy and surgery	3
Hormone therapy and chemotherapy	2
Radiotherapy, chemotherapy, and surgery	2
Radiotherapy and chemotherapy	2
Only chemotherapy	6
Only hormone therapy	2
Only radiotherapy	1
No treatment	2
Not reported	5
Total	29

Breast cancer stage	Number of patients
Stage 0	1
Stage I	1
Stage IIA	1
Stage IIIA	1
Stage IIB	3
Stage IIIB	2
Not reported	20
Total:	29

Although our original aim was to recruit females who had recently been diagnosed with breast cancer and were at early stages, our sample is heterogeneous. Specific information regarding the breast cancer stage was only available for a subset of the participants (as reported in Table 1 above)

The control group was composed of 29 healthy participants. The patients and controls were matched in age and years of formal education (Table 2). For the controls, the inclusion criteria were healthy adult females from the same geographical region as the patients. The exclusion criteria were a history of previous cancer, current neurological illness, psychiatric disorder, or substance abuse. For the patients, the inclusion criteria were adult females recently diagnosed with breast cancer. The exclusion criteria were history of other type of cancer, neurological illness, psychiatric disorder, or substance abuse.

**Table 2:** Summary of the demographic data

	Patients *(n* = *29)*	Controls *(n* = *29)*	Test statistic	Patients vs controls
	Mean (SD)	Mean (SD)		P-value
Age (years)ª	51.655 (12.036)	50.655 (11.806)	− 0.319	0.751
Years of education^b^	12 (3.827)	13.759 (4.509)	521	0.117

*SD* standard deviation

^a^p values were calculated through Student’s t-test

^b^p values were calculated through Mann–Whitney U-test

^*^Alpha level set at .05

## Instruments

### Social cognition

#### Emotion recognition

We used a short version of the Emotion Evaluation Test (EET). The EET is the first part of The Awareness of Social Inference Test (TASIT) [46]. We measured the recognition of basic emotional expressions (disgust, fear, surprise, sadness, and anger) through a subset of 10 short videos. After watching the video, the participant had to identify the emotion that illustrated what the character was feeling. We obtained a score for each emotion identified correctly and added the correct answers to obtain the total score. Based on previous studies, the TASIT has a good test–retest reliability [47]. The Cronbach alpha for our dataset was α = 0.69. It should be noted that this test has been previously used in numerous studies [48–54] to measure emotion recognition in clinical and non-clinical populations.

#### Theory of mind (ToM)

The affective component of ToM was assessed through the Reading the Mind in the Eyes test (RMET) [55, 56]. In this case, there are 36 photographs showing the eyes of people with different emotional states. The participant is presented with four possible options, and she has to select what the person in the image is probably feeling. We compared the total number of correct answers. Although the reported Cronbach alpha for the RMET typically ranges from 0.48 to 0.63 [57, 58], it has good test–retest reliability [59], and it has been adapted to Spanish [60]. The RMET has been used to assess ToM in previous studies in Latin America [48–50, 61]. In our dataset, we had a good internal consistency, estimated by a Cronbach alpha of approximately α = 0.853.

#### Empathy

The Interpersonal Reactivity Index (IRI) [62] measures cognitive and emotional components of empathy through self-report. Each item was rated 0 to 4 so that the maximum score was 112 points. The instrument is composed of four seven-item subscales: Perspective-taking, Fantasy, Personal distress, and Empathic concern. Perspective-taking and Fantasy refer to a cognitive component. Perspective-taking measures the ability to consider other people’s points of view, whereas fantasy assesses the propensity to identify with fictional characters. On the other hand, personal distress and empathic concern emphasize the affective component of empathy. Personal distress measures if the participant reports feeling discomfort when they witness someone else experience a negative situation. Finally, the empathic concern subscale indicates if the participant feels compassionate and concerned towards other people [62]. Based on previous studies, the Cronbach alpha for the IRI subscales typically range from 0.56 to 0.80 [63, 64]. In our dataset, our internal consistency was relatively similar to the one reported in the literature. Indeed, the approximate Cronbach alpha for fantasy was 0.657, for empathic concern was 0.703, for personal distress was 0.496, and for perspective taking was 0.567. This task has been previously applied as an empathy measure in Latin American countries [50, 65–67].

#### Moral emotions

The Moral Sentiment Association Task—MSAT [68] describes different possible situations and the participant has to select the emotion they would likely feel if they were in that scenario. An adapted Spanish version of the task was shown to have acceptable internal consistency in Chile of α = 0.79 [69]. In our case, we used an abbreviated version including 28 situations and the Cronbach alpha for the task was estimated as α = 0.567, which allowed us to have measures across different types of emotions in neutral, moral, and non-moral scenarios. In this task, the participant is presented with a statement describing the situation and four options of possible emotions. For example, a statement meant to evoke disgust is: “Your neighbor came to help you wash your car. When you looked at the engine you saw pieces of a dead rat” [68]. If the participant reports not feeling an emotion or if the emotion they feel was not included in the list, they were instructed to select the “neutral” option. We counted the number of times that a participant selected an emotion that accurately matched the social situation and calculated the percentage of correct answers. We analyzed each emotion separately and we also grouped them into two categories: On one group, we compiled the items that were meant to directly evoke basic emotions of fear or disgust. On the other group, we included the situations that had a greater social “moral” component. Using a scale from 1 to 10, the participants also reported the intensity of the emotion and its impact.

### General cognition

The Montreal Cognitive Assessment—MoCA [70] and the INECO Frontal Screening—IFS [71] were used as measurements of general cognitive function. The MoCA subdivides into several tasks of visuospatial and executive functions, naming, attention, concentration and calculation, language, abstractions, delayed recall, and orientation [70]. The IFS measures several executive function domains. It subdivides into the following tasks: Luria motor series, conflicting instructions, motor inhibitory control, proverb interpretation, modified Hayling test for verbal inhibitory control, backward digits span, months backward, and modified Corsi tapping test [71]. Based on previous studies, the Cronbach alpha for MoCA is around 0.83–0.85 [70, 72], whereas the Cronbach alpha for IFS is typically around 0.8–0.9 [71, 73].

### Clinical and socio-emotional measures

#### Quality of life

The EORTC QLQ-C30 questionnaire measures different aspects of quality of life. It was originally designed for clinical trials in oncology [74] and it has also been used and validated in Colombia [75, 76]. Based on previous studies, the reported Cronbach alpha for the EORTC QLQ-C30 subscales is generally above 0.7 [77, 78], although some subscales can have a lower score (e.g., α = 0.52 for the cognitive subscale) [78]. This self-report questionnaire includes a symptom scale, a global assessment score (general health and quality of life rated from 1 to 7), and several functional scales (including physical, cognitive, social, role, and emotional components). Items regarding physical functioning were coded as a dichotomous yes/no question. The items for the other functional scales were rated from 1 to 4. As suggested in the scoring manual, we did linear transformations to obtain the scores in a range of 100 points [79].

#### Depression

We applied the Zung Depression Scale as a self-report measure of the depressive symptoms experienced in the last few days [80, 81]. The questionnaire includes items for emotional, somatic, and cognitive symptoms. We compared the SDS index among groups. Based on previous studies, the Zung Depression Scale has been validated and typically shows a good internal consistency, estimated by a Cronbach alpha ranging from 0.84 to 0.89 [82–84].

#### Anxiety

The GAD-7 was developed as an instrument to assess generalized anxiety disorder [85]. This self-report questionnaire has 7 items and the final score ranges between 0 and 21, where higher values indicate increased severity of anxiety symptoms. Based on previous studies, the GAD-7 has been validated, shows good test–retest reliability, and typically has great internal consistency, estimated by a Cronbach alpha around 0.92–0.93 [85–87].

### Statistical analysis

We revised the assumptions of normality and variance homoscedasticity with Shapiro–Wilk and Levene’s test, respectively. Depending on the outcome, we compared the groups with the independent sample t-test, Welch t-test, or Mann–Whitney U test. The alpha threshold was initially set at 0.05. However, we also reported results that remained significant after the Bonferroni correction. For the independent t-test and Welch test, the effect size was given by Cohen’s d, whereas Rank-Biserial Correlation (r_B_) was used for the Mann–Whitney U test. Finally, we performed linear multiple regressions to predict the social cognition variables that differed between groups (EET, RMET, MSAT). The predictors were the group (patients or controls), GAD-7 scores, emotional and social subscales of EORTC QLQ-C30, and IFS scores. To explore the role of age and education on social cognition, we also included other models, where we added age and years of study as additional predictors. Statistical analyses were carried out in JASP 0.14.1.0 [88]. We used SPSS version 28 [89] for the Cronbach alpha calculations for EET, RMET, IRI, and MSAT.

## Results

### Social cognition

**Fig. 1:**
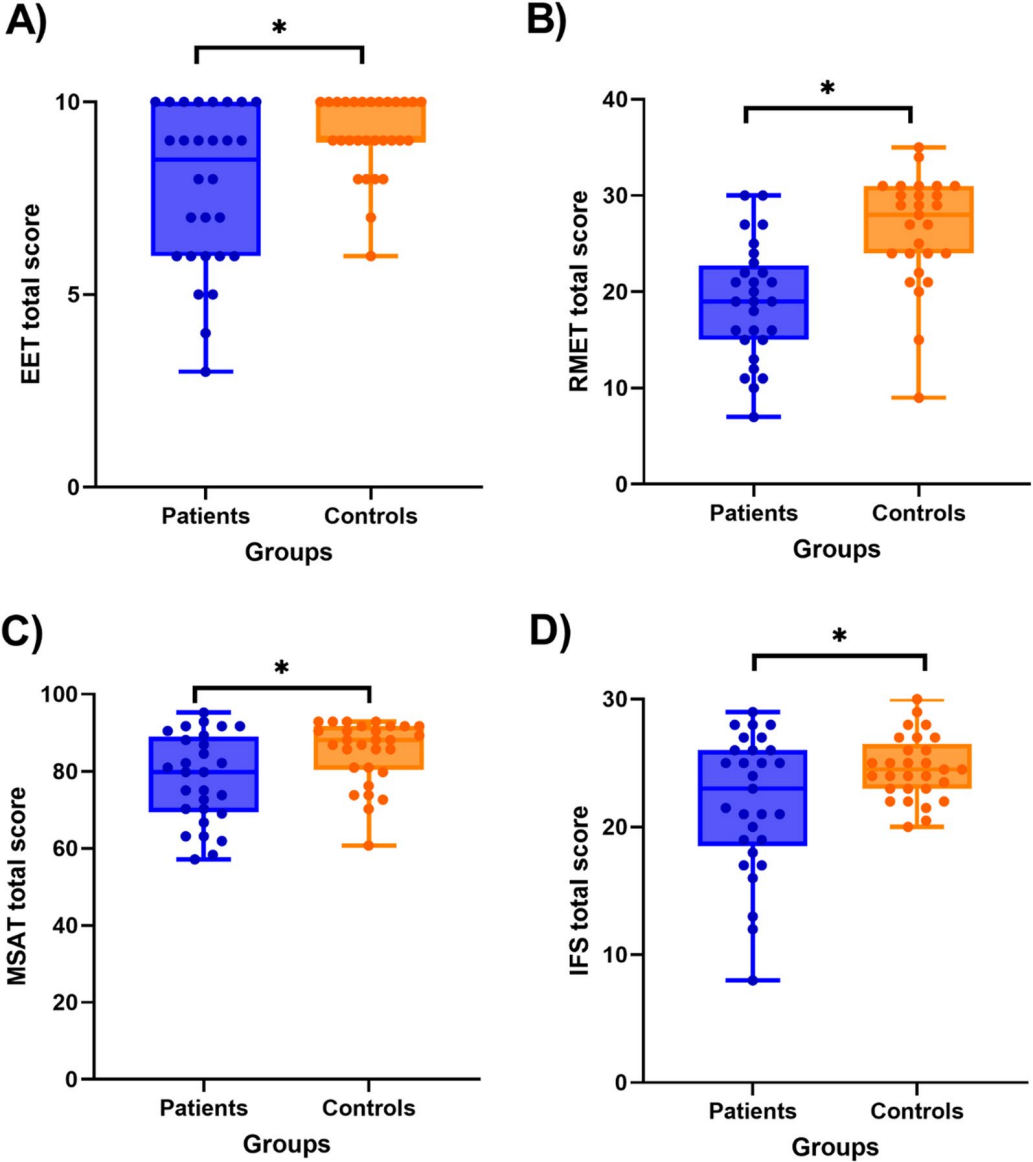
EET, Emotion Evaluation Test; IFS, INECO Frontal Screening; MSAT, Moral Sentiment Association Task; RMET, Reading the Mind in the Eyes Test. Boxplots for the comparison of executive functions and social cognition tasks. **A** Patients have lower accuracy in the EET (p = 0.016). **B** Patients have lower scores in the RMET (p < 0.001). **C** Patients have a lower performance in the MSAT (p = 0.021). **D** Patients have lower scores in the IFS (p = 0.018)

Based on the EET total score, the patients had a decreased ability to correctly recognize emotions (U = 551, p = 0.016, r_B_ = 0.357) (Fig. 1A). When we compared the accuracy for each emotion separately (Additional file 1: Table S1), we found that patients had lower scores in videos that depicted disgust (U = 572.5, p = 0.011, r_B_ = 0.361).

Patients also had a worse performance in the RMET task for ToM (U = 623, p < 0.001, r_B_ = 0.648) (Fig. 1B). However, there were no significant differences in the total IRI score for empathy (t_(54)_ = 1.081, p = 0.285, d = 0.289) nor in any of the IRI subscales (Additional file 1: Table S1).

In the moral sentiment task, patients had an overall lower accuracy than controls (U = 551, p = 0.021, r_B_ = 0.357) (Fig. 1C). Nevertheless, we did not find significant differences in the emotional intensity or impact (Additional file 1: Table S2). When we analyzed each emotion separately, we found differences in the correct attribution of neutral situations (U = 509, p = 0.024, r_B_ = 0.254). In neutral situations, controls correctly reported feeling neutrality more often than patients. There were similar trends for other emotions, like anger or pity (Additional file 1: Table S2). We also grouped MSAT items depending if the situation had a social moral implication (“moral”) or not (“basic” emotions of fear and disgust). However, when we compared patients and controls, we did not find significant differences in their performance within those categories (Additional file 1: Table S2).

It should be noted that the difference in ToM (RMET scores) was the only result that remained significant if we applied the threshold of the new significance cut-off after Bonferroni correction [90, 91].

### General cognition

There were no significant differences between patients and controls in the total score of the MoCA (U = 486.5, p = 0.306, r_B_ = 0.157), nor any of the MoCA subscales. However, in measures of executive functions, patients had a lower performance than controls in the IFS total score (t = 2.459, p = 0.018, d = 0.646) (Fig. 1D). When analyzing the IFS subscales, patients had a lower performance in the tasks of conflicting instructions (U = 509.5, p = 0.022, r_B_ = 0.212), and verbal inhibitory control (U = 580, p = 0.009, r_B_ = 0.379) (Additional file 1: Table S3). These results were not significant if we applied the new threshold of Bonferroni correction [90, 91].

### Socioemotional measures

**Fig. 2:**
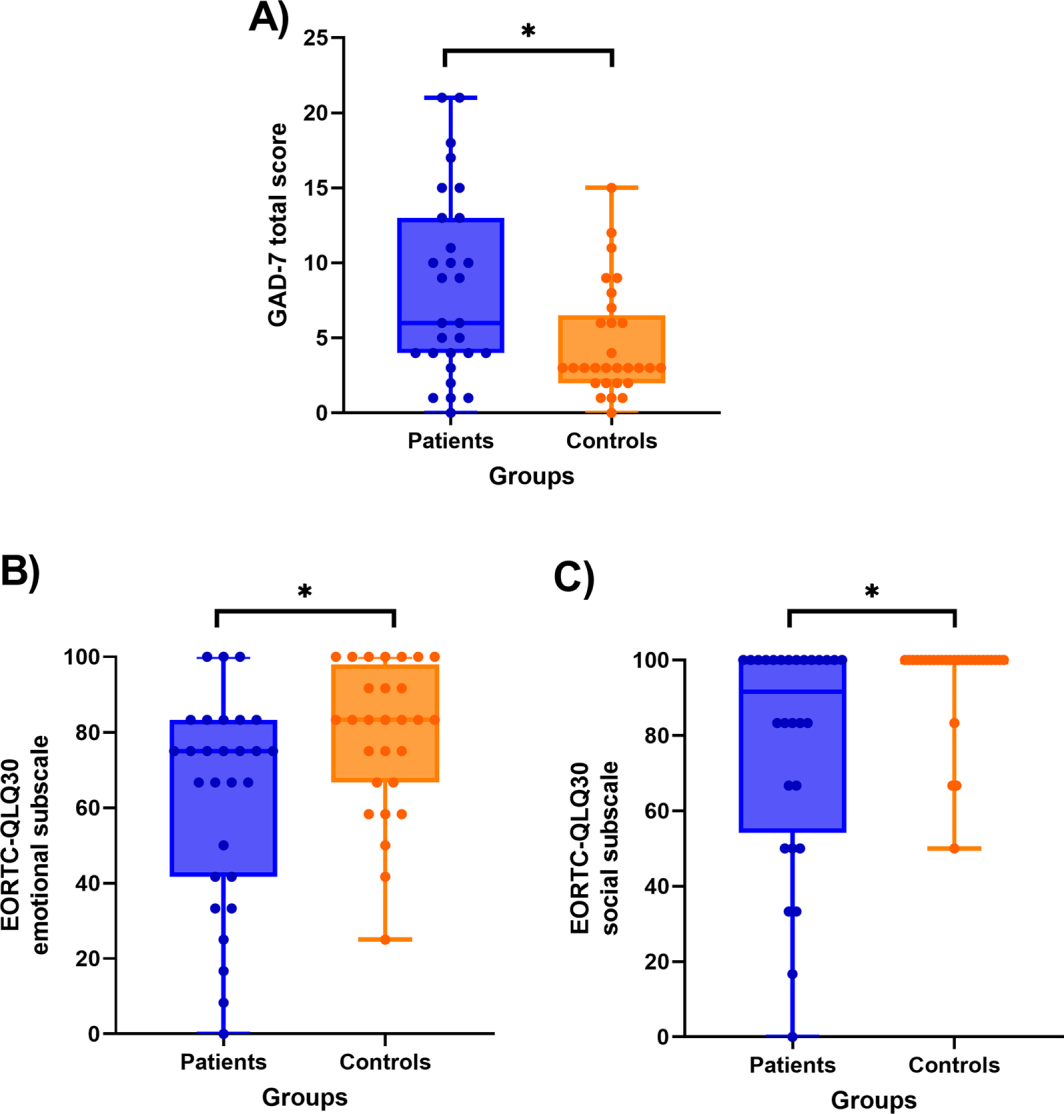
EORTC QLQ-C30, European Organisation for Research and Treatment of Cancer Quality of Life Questionnaire Core 30; GAD-7, Generalized Anxiety Disorder Questionnaire. Boxplots for the comparison in socioemotional measures. **A** Patients report higher levels of anxiety (p = 0.012). **B** Patients have a lower level of functioning within the emotional functioning subscale of the EORTC QLQ-C30 (p = 0.018). **C** Patients have a lower level of functioning within the social functioning subscale of the EORTC QLQ-C30 (p = 0.003)

The patients reported higher levels of anxiety in the GAD-7 questionnaire (U = 259, p = 0.012, r_B_ = − 0.384) (Fig. 2A). Also, we found differences in some of the functional subscales of EORTC QLQ-C30 (Additional file 1: Table S4). In fact, the emotional (U = 535.5, p = 0.018, r_B_ = 0.366) and social (U = 558, p = 0.003, r_B_ = 0.374) subcomponents scores were lower in patients than in controls (Figs. 2B, C). However, in the SDS index measure of depressive symptoms, patients and controls were not significantly different (t_(55)_ =  − 1.345, p = 0.184, d = − 0.356). These results were not significant if we applied the new threshold of Bonferroni correction [90, 91].

### The relationship between social cognition and relevant cognitive and socioemotional variables

We conducted three multiple regressions to predict the social cognition measures in which we found significant differences (EET, RMET, and MSAT). As predictors, we selected the cognitive and socioemotional variables that differed between patients and controls, such as GAD-7 scores, the emotional and social subscales of EORTC QLQ-C30, and the IFS scores. The group (patients or controls) was also included as a predictor in all models.

The model including emotion recognition (EET score) as a dependent variable (F_(5, 49)_ = 14.574, p < 0.001, adjusted R^2^ = 0.557) showed that group (standardized β = − 0.285, t = − 2.604, p = 0.012) and IFS score (standardized β = 0.624, t = 6.25, p < 0.001) were significant predictors. In the model where ToM (RMET score) was the dependent variable (F_(5, 47)_ = 6.216, p < 0.001, adjusted R^2^ = 0.334), the group (standardized β = − 0.497, t = − 3.62, p < 0.001) was still a significant predictor. However, the IFS score approached significance but did not reach the threshold (standardized β = 0.245, t = 1.949, p = 0.057). Finally, the model including MSAT accuracy as the dependent variable was not significant and had a low adjusted R^2^ of 0.099 (F_(5, 49)_ = 2.185, p = 0.071).

We also re-analyzed the models after including age and years of study as additional predictors. In the model where emotion recognition (EET score) was the dependent variable (F_(7, 47)_ = 10.596, p < 0.001, adjusted R^2^ = 0.554), the group (standardized β = − 0.262, t = − 2.329, p = 0.024) and executive functioning—IFS (standardized β = 0.595, t = 4.899, p < 0.001) were still significant predictors. Age and years of study did not reach significance. In the model where ToM (RMET) was the dependent variable (F_(7, 45)_ = 6.208, p < 0.001, adjusted R^2^ = 0.412), the group (standardized β = − 0.444, t = − 3.365, p = 0.002) was still a significant predictor, as well as years of education (standardized β = 0.347, t = 2.676, p = 0.01). The model for the moral emotions task (MSAT) was not significant even after adding age and years of study (F_(7,47)_ = 1.562, p = 0.17, adjusted R^2^ = 0.068).

## Discussion

This study compared different social cognition domains in female breast cancer patients and healthy controls. Our results showed that patients exhibited impairments in emotion recognition, ToM, and some measures of executive function. This study contributes to a better understanding of the social challenges in breast cancer patients, pointing toward the underlying problems in emotion-related social cognition domains.

### Social cognition

We found that patients had impairments in some social cognition tasks. Indeed, they had problems in emotion recognition, particularly with disgust. In this case, the limited number of items per emotion category limits the interpretation. Nevertheless, considering the patient’s impairments in executive functions and ToM, they might wrongly identify the emotional reactions of others by focusing on their experiences instead of other people’s cues. Previously, different studies had analyzed emotional expression and regulation in breast cancer [92], but the effects on emotion recognition had not been fully explored. Although the study of Graves et al. [93] did not find differences in emotion recognition, they detected some minor changes in emotional expression. It is possible that these results are different from the current ones due to variations in the task nature or due to distinct sample characteristics since Graves et al. [93] recruited breast cancer survivors that had already completed therapy. Future studies should investigate the trajectory of changes in emotion recognition before and after treatment.

We also found that patients had a lower performance in affective ToM and moral emotions tasks. The impairment in ToM is consistent with the results of previous studies, where patients with breast cancer also had a lower performance in RMET [5–7]. Considering the relationship between emotion recognition and affective ToM, a deficit in both of these domains may be interconnected and lead to greater alterations. This relationship is expected because ToM and emotion recognition implies the recruitment of similar brain regions [35]. Indeed, the performance at the RMET task could also be partly explained by deficits in emotion recognition. Furthermore, it is possible that the difficult self-centered emotional experiences involving cancer are either demotivating or biasing the response when the patients try to infer the mental states of other people. Regarding the moral sentiment task, patients and controls differed in their attribution of neutrality. Thus, it is possible that patients are having different emotional reactions to the hypothetical scenarios presented in the MSAT where neutral situations are actually evoking an emotional response. Considering that, in our dataset, the abbreviated MSAT had a Cronbach alpha lower than desired, future studies should assess the potential alterations in moral and non-moral scenarios using more extensive MSAT items, as well as additional psychometric measures for moral emotions. More research is needed to explore the attribution of emotional labels specifically in moral situations.

Previous studies have shown that patients with brain tumor and patients in remission from primary CNS lymphoma had impairments in some social cognition measures [36, 94]. However, it is interesting that cancer outside the CNS can also lead to social cognition alterations. For example, patients with esophageal cancer and depression also have ToM impairments [95]. Although most of our results did not withstand the Bonferroni correction, the difference in the ToM task remained significant. This indicates that ToM seems to be the more impaired social cognition domain in breast cancer females. Regarding the potential mechanisms, Pertz et al. suggested possible explanations for socio-cognitive deficits in cancer (primary CNS lymphoma in remission), including an alteration in the motivational state of patients when they are facing a potentially fatal disease [36].

Overall, our results showed that patients with breast cancer undergoing different treatments may be facing social cognition challenges, which could potentially affect their interactions with others. This is particularly relevant because social cognition domains can help predict mental illness symptoms [96]. Moreover, social support is important for the quality of life of breast cancer patients [38] and a declining quality of social support can be related to more stress and depression [40]. Thus, an impaired ability to recognize other people’s emotions or identify their mental states could jeopardize the quality of interactions with others and this, in turn, could worsen their own emotional alterations.

### General cognition

Regarding general cognitive measures, we found that patients showed impairments in executive functions, specifically in verbal inhibitory control and conflicting instructions. Although previous studies have found deficits in executive functions in females treated with chemotherapy [31], these impairments are not always consistent, partially due to the variety of tests to assess them. It has been proposed that chemotherapy could mainly affect tasks measuring updating and shifting [97]. Further studies are needed to determine the extent of specific deficits in some domains, while still preserving others.

We did not detect differences between patients and controls in the MoCA assessment for general cognitive functioning. Previous analyses have indicated that breast cancer treatments may compromise some cognitive domains [22, 23, 25]. Both genetic predispositions and socio-demographic factors can help account for the risk of developing cancer-related cognitive impairment [10]. It is also possible that the heterogeneity in treatments and cancer stages prevented a clearer detection of the physical and cognitive alterations in our sample. Our result showing non-significant differences between groups may also be associated with the chosen measures and future studies should assess more extensive neuropsychological batteries. However, it should be noted that patients can have different cognitive trajectories and not all of them decline [98]. Thus, differences in basic cognition are not always found [99, 100] and, although several patients report cognitive alterations [101], some long-term breast cancer survivors do not significantly differ from the normative group in regards to cognitive complaints [102]. It is also important to note that objective impairments and subjective cognitive complaints are not always consistently related [103].

### The relationship between social cognition and relevant cognitive and socioemotional variables

Previous research has found that breast cancer patients commonly suffer from depression and anxiety [2]. In this study, although patients and controls did not significantly differ in depressive symptoms, patients reported higher anxiety in the GAD-7 and lower socioemotional functioning in the EORTC QLQ-C30. Nevertheless, contrary to what we originally predicted, these emotional variables did not fully account for the social cognition alterations. Indeed, in the multiple regressions, the anxiety and the EORTC QLQ-C30 socioemotional subscales were not significant predictors. Although other studies have suggested that breast cancer patients with depression have more severe ToM deficits [6], it is possible that this relationship depends on the baseline characteristics of the sample, since our group of patients did not have significantly higher depression scores.

Regarding the relationship between social and general cognition, we found that the IFS was a predictor for the EET task but did not reach significance for the RMET task. This indicates that, in the population of study, executive functions can be related to some domains of social cognition, which may be due to a partial overlap of brain areas relevant to both processes. Previous studies have also found relationships between executive functions and emotion recognition in fibromyalgia [104]. Overall, although general cognition can be related to social cognition deficits, it would not fully account for the impairments [105].

We also found that years of education was a significant predictor of ToM. This is consistent with previous studies showing a better ToM performance in people with more education [106]. It should be noted that the group category of controls versus patients remained a significant predictor even after accounting for age, years of study, executive functions (IFS scores), and emotional alterations (GAD-7 and EORTC QLQ-C30 socioemotional subscales). This suggests that the effects of breast cancer on social cognition are not fully explained by the included measures. Therefore, future studies should analyze whether some of the social cognition alterations could perhaps be a direct effect of cancer and its treatments or if the patient’s motivation to engage in social cognition tasks is partially responsible.

It is important to note that we only focused on female patients due to the high prevalence of breast cancer in that population [1]. However, there might be sex differences between males and females in social cognition domains. A study by Di Tella et al. [107] found that females had higher empathy scores in some IRI subscales and better accuracy for anger recognition. However, there were not significant differences in the recognition of other emotions or in ToM scores. The phase of the menstrual cycle (follicular or luteal) did not have a significant effect either [107]. Nevertheless, these sex differences may be impacted by the chosen self-report measures and the gender stereotypes. A study analyzing empathy for pain showed that the effects sizes for sex differences are very small. Thus, sex was a great predictor for self-report measures (like IRI) but not for empathy for pain [65]. Future analyses should explore the effect of menopause and sex-differences in the social cognition abilities of cancer patients.

There are limitations considering the exploratory nature of this study. We wanted to characterize the effects of breast cancer in social cognition without a specific emphasis on a certain treatment avenue. However, due to the diversity of treatments, we cannot discriminate the effect of cancer from the effect of treatment. Similarly, our sample size limited the effect sizes that we could reliably detect and precluded the comparisons between treatments and stages. Another limitation is that we only had available data of the breast cancer stage for a subset of participants. Therefore, we cannot confidently extrapolate these findings to specific stages or analyze whether the invasiveness and spread of the cancer could have an impact. We recognize that our sample is heterogeneous in regards to treatment and stages. However, considering that we detected some preliminary social cognition alterations in this exploratory study, it is possible that clearer patterns could be found by focusing only in specific advanced stages. In addition, considering that most of our results did not remain significant after the Bonferroni correction, future studies should replicate these findings with a larger sample size that allows comparisons amongst treatments and includes neuroimaging measures. Moreover, longitudinal studies that track social cognition before and after treatment would help clarify causality and account for baseline differences. We found that breast cancer patients can experience impairments in some domains of social cognition, which is important due to the incidence of breast cancer [1] and the role of social support in emotional well-being [40]. In regards to clinical implications, this could indicate the need for therapeutic interventions to preserve social cognition skills in patients, which could hopefully improve their support system when facing cancer. Furthermore, if these findings are replicated, it could be relevant to advise the patients and their close ones about possible social cognition changes so that we could promote a more compassionate understanding in their support system. This could be particularly beneficial considering the impact of support on quality of life [38] and the mental health problems associated with low-quality support [40].

## Conclusion

In conclusion, this study contributed to identifying social cognition deficits in breast cancer patients with various treatments. Even considering the heterogeneity of the sample, we identified deficits in emotion recognition and ToM. The patients also experienced lower performance in some executive functions, increased anxiety, and more self-reported socioemotional alterations. Overall, our results suggest that social cognition domains are relevant variables to consider when addressing female breast cancer.

## Supplementary Information

**Additional file 1**. Report of means, standard deviations, and statistical comparisons between patients and controls.

## Data Availability

The datasets used and/or analyzed during the current study are available from the corresponding author on reasonable request.

## Abbreviations

EET: Emotion evaluation test
IFS: INECO frontal screening
MSAT: Moral sentiment association task
TASIT: The awareness of social inference test
ToM: Theory of mind
RMET: Reading the mind in the eyes test
