# Supplementary material for: The impact of breast cancer on social cognition in female Colombian patients
Source: BMC Psychol. 2022 Dec 13;10:303. doi: 10.1186/s40359-022-01005-1 (PMC9745936; doi:10.1186/s40359-022-01005-1)
Supplement: Supplementary file 1 — Additional file 1. Report of means, standard deviations, and statistical comparisons between patients and controls. [file 40359_2022_1005_MOESM1_ESM.docx]

**Supplementary Table 1.** Social cognition assessment: Analysis of EET, IRI, and RMET

|  | Patients *(n=29)* | Controls *(n=29)* | Test statistic | Patients vs controls |
| --- | --- | --- | --- | --- |
|  | Mean (SD) | Mean (SD) |  | P-value |
| EET^b^ | 7.786 (2.097) | 9.138 (1.026) | 551 | 0.016* |
| EET disgust^b^ | 1.00 (0.845) | 1.552 (0.632) | 572.5 | 0.011* |
| EET anger^b^ | 1.759 (0.511) | 1.931 (0.258) | 479.5 | 0.128 |
| EET fear^b^ | 1.679 (0.548) | 1.862 (0.351) | 468 | 0.165 |
| EET surprise^b^ | 1.828 (0.468) | 1.966 (0.186) | 464.5 | 0.164 |
| EET sadness^b^ | 1.552 (0.632) | 1.828 (0.384) | 512.5 | 0.068 |
| IRI Total scoreª | 59.75 (12.58) | 63.571 (13.85) | 1.081 | 0.285 |
| Perspective-takingª | 15.786 (5.181) | 16.821 (4.514) | 0.798 | 0.429 |
| Fantasyª | 11.931 (5.496) | 14.034 (6.428) | 1.339 | 0.186 |
| Empathic concern ^b^ | 18.379 (5.71) | 19.966 (5.454) | 476.5 | 0.387 |
| Personal distressª | 14.25 (4.461) | 12.536 (5.203) | -1.324 | 0.191 |
| RMET^b^ | 18.929 (6.012) | 26.37 (5.805) | 623 | <0.001* |

Abbreviations: EET TASIT, Emotion Evaluation Test from The Awareness of Social Inference Test; IRI, Interpersonal Reactivity Index; RMET, Reading the Mind in the Eyes Test

^a^p values were calculated through Student’s t-test

^b^p values were calculated through Mann-Whitney U-test

*Alpha level set at .05

**Supplementary Table 2.** Analysis of the situations meant to evoke an emotion (measured as percentage of correct answers), as well as its intensity and impact, in the Moral Sentiment Association Task (MSAT).

|  | Patients *(n* = 29*)* | Controls *(n* = 29*)* | Test  statistic | Patients vs controls |
| --- | --- | --- | --- | --- |
|  | Mean (SD) | Mean (SD) |  | P-value |
| Embarrassment^b^ | 68.75  (27.743) | 79.31  (25.937) | 498.5 | 0.122 |
| Intensity^a^ | 7.786  (1.641) | 7.819  (1.392) | 0.083 | 0.934 |
| Impact^a^ | 7.188  (1.709) | 7.138  (1.571) | -0.114 | 0.91 |
| Fear^b^ | 81.25  (21.109) | 90.517  (14.037) | 501.5 | 0.087 |
| Intensity^b^ | 8.196  (1.585) | 8.276  (1.24) | 406 | >0.99 |
| Impact^b^ | 7.92  (1.827) | 7.759  (1.418) | 355 | 0.419 |
| Disgust^b^ | 77.976  (24.867) | 79.31  (17.624) | 388.5 | 0.778 |
| Intensity^a^ | 5.815  (1.965) | 6.569  (1.872) | 1.483 | 0.144 |
| Impact^a^ | 4.952  (1.964) | 5.185  (2.018) | 0.436 | 0.664 |
| Neutral^b^ | 91.071  (12.199) | 97.414  (7.748) | 509 | 0.024* |
| Intensity^b^ | 3.295  (3.656) | 2.44  (2.68) | 371 | 0.535 |
| Impact^b^ | 3.277  (3.662) | 1.422  (0.887) | 315 | 0.085 |
| Pity/compassion^b^ | 87.5  (15.957) | 93.966  (14.416) | 503 | 0.055 |
| Intensity^b^ | 7.982  (1.7) | 8.629  (1.051) | 479.5 | 0.242 |
| Impact^c^ | 7.545  (1.916) | 8.198  (1.142) | 1.557 | 0.127 |
| Guilt^b^ | 66.667  (30.089) | 68.966  (26.624) | 421.5 | 0.799 |
| Intensity^a^ | 7.333  (1.757) | 7.816  (1.736) | 1.044 | 0.301 |
| Impact^a^ | 6.976  (1.692) | 7.506  (1.62) | 1.207 | 0.233 |
| Anger  /indignation^b^ | 72.619  (25.746) | 85.057  (22.863) | 517 | 0.051 |
| Intensity^a^ | 6.845  (1.945) | 7.598  (1.78) | 1.525 | 0.133 |
| Impact^a^ | 6.393  (2.124) | 6.586  (2.068) | 0.348 | 0.729 |
| Total basic emotions of fear and disgust^b^ | 79.613  (16.131) | 84.914  (10.352) | 460 | 0.389 |
| Intensity^a^ | 7.006  (1.586) | 7.422  (1.375) | 1.06 | 0.294 |
| Impact^a^ | 6.436  (1.709) | 6.472  (1.479) | 0.084 | 0.934 |
| Total “moral” scenario emotions^b^ | 73.884  (16.32) | 81.825  (12.248) | 516.5 | 0.077 |
| Intensity^a^ | 7.487  (1.399) | 7.966  (1.11) | 1.434 | 0.157 |
| Impact^a^ | 7.025  (1.525) | 7.357  (1.147) | 0.931 | 0.356 |
| Total emotions^b^ | 77.976  (11.471) | 84.934  (8.28) | 551 | 0.021* |
| Intensity^a^ | 6.75  (1.29) | 7.021  (1.114) | 0.848 | 0.4 |
| Impact^a^ | 6.321  (1.492) | 6.262  (1.048) | -0.173 | 0.863 |

Abbreviations: MSAT, Moral Sentiment Association Task

^a^p values were calculated through Student’s t-test

^b^p values were calculated through Mann-Whitney U-test

^c^p values were calculated through Welch’s t-test.

*Alpha level set at .05

**Supplementary table 3.** General Cognition assessment: Analysis of the subscales of MoCA and IFS

|  | Patients  *(n* = 29*)* | Controls  *(n* = 29*)* | Test statistic | Patients vs controls |
| --- | --- | --- | --- | --- |
|  | Mean (SD) | Mean (SD) |  | P-value |
| MOCA^b^ | 24.828 (4.132) | 26.207 (2.704) | 486.5 | 0.306 |
| MOCA visuospatial^b^ | 3.966 (1.180) | 4.172 (1.037) | 456.5 | 0.555 |
| MOCA naming^b^ | 2.724 (0.591) | 2.828 (0.384) | 440 | 0.665 |
| MOCA attention^b^ | 4.724 (1.601) | 5.207 (1.082) | 477 | 0.345 |
| MOCA language^b^ | 2.276 (0.922) | 2.517 (0.634) | 465 | 0.442 |
| MOCA abstraction^b^ | 1.793 (0.559) | 1.69 (0.604) | 367.5 | 0.275 |
| MOCA memory^b^ | 3.517 (1.455) | 3.69 (1.228) | 439.5 | 0.766 |
| MOCA orientation^b^ | 5.828 (0.468) | 5.966 (0.186) | 464.5 | 0.164 |
| IFS^c^ | 21.914 (5.345) | 24.603 (2.476) | 2.459 | 0.018* |
| IFS motor series^b^ | 2.897 (0.31) | 2.828 (0.384) | 391.5 | 0.458 |
| IFS conflicting instructions^b^ | 2.586 (0.825) | 2.966 (0.186) | 509.5 | 0.022* |
| IFS motor inhibitory control^b^ | 2.138 (1.187) | 2.621  (0.677) | 500 | 0.146 |
| IFS backward digits span^b^ | 3.621 (1.613) | 4.034 (1.085) | 471 | 0.424 |
| IFS months backward^b^ | 1.759 (0.435) | 1.931 (0.258) | 493 | 0.074 |
| IFS visuospatial working memory^b^ | 2.276 (1.131) | 2.552 (0.948) | 473 | 0.401 |
| IFS proverb interpretation^b^ | 2.638 (0.667) | 2.431 (0.651) | 322 | 0.085 |
| IFS verbal inhibitory control^b^ | 4  (1.946) | 5.241 (0.689) | 580 | 0.009* |

Abbreviations: IFS, INECO Frontal Screening; MOCA, Montreal Cognitive Assessment

^a^p values were calculated through Student’s t-test

^b^p values were calculated through Mann-Whitney U-test

^c^p values were calculated through Welch’s t-test.

*Alpha level set at .05

**Supplementary Table 4.** Socioemotional measures: Analysis of EORTC QLQ-C30, SDS-Zung, and GAD-7.

|  | Patients *(n* = 29*)* | Controls *(n* = 29*)* | Test statistic | Patients vs controls |
| --- | --- | --- | --- | --- |
|  | Mean (SD) | Mean (SD) |  | P-value |
| Physical functioning ^b^ | 90  (14.907) | 88.966 (15.663) | 394 | 0.83 |
| Role  subscale ^b^ | 91.071 (13.211) | 90.805 (18.68) | 441.5 | 0.503 |
| Cognitive subscale ^b^ | 71.429 (27.162) | 81.034 (20.278) | 483.5 | 0.201 |
| Emotional subscale ^b^ | 62.798 (27.637) | 78.869 (19.705) | 535.5 | 0.018* |
| Social subscale ^b^ | 77.976 (29.416) | 95.402 (12.517) | 558 | 0.003* |
| Symptoms scale^b^ | 24  (18.738) | 14.927 (13.694) | 248 | 0.07 |
| Global Health Status/QoL ^b^ | 80.655 (13.991) | 82.44 (13.483) | 416 | 0.693 |
| Zung SDS index^a^ | 45.938 (10.294) | 41.94 (12.048) | -1.345 | 0.184 |
| GAD-7^b^ | 8.345  (6.166) | 4.621  (3.678) | 259 | 0.012* |

Abbreviations: EORTC QLQ C-30, Questionnaire developed to assess the quality of life of cancer patients; GAD-7, Generalized Anxiety Disorder Questionnaire; SDS Zung, Zung Self-Rating Depression Scale SDS index score

^a^p values were calculated through Student’s t-test

^b^p values were calculated through Mann-Whitney U-test

*Alpha level set at .05
